# Plant Occurring Flavonoids as Modulators of the Aryl Hydrocarbon Receptor

**DOI:** 10.3390/molecules26082315

**Published:** 2021-04-16

**Authors:** Elizabeth Goya-Jorge, María Elisa Jorge Rodríguez, Maité Sylla-Iyarreta Veitía, Rosa M. Giner

**Affiliations:** 1 Departament de Farmacologia, Facultat de Farmàcia, Universitat de València. Av. Vicente Andrés Estellés, s/n, Burjassot, 46100 Valencia, Spain; egojor@alumni.uv.es; 2 ProtoQSAR SL., CEEI (Centro Europeo de Empresas Innovadoras), Av. Benjamin Franklin 12, Parque Tecnológico de Valencia, Paterna, 46980 Valencia, Spain; 3 Departamento de Farmacia, Facultad de Química-Farmacia, Universidad Central “Marta Abreu” de las Villas, C. Camajuaní km 5½, Santa Clara 54830, Villa Clara, Cuba; elisa@uclv.edu.cu; 4 Equipe de Chimie Moléculaire du Laboratoire Génomique, Bioinformatique et Chimie Moléculaire (EA 7528), Conservatoire National des Arts et Métiers (Cnam), 2 Rue Conté, HESAM Université, 75003 Paris, France; maite.sylla@lecnam.net

**Keywords:** flavonoids, phytochemicals, phytocompounds, Ah receptor, dioxin receptor, polyphenols, transcription factor, bioactive, xenobiotics, functional food

## Abstract

The aryl hydrocarbon receptor (AhR) is a transcription factor deeply implicated in health and diseases. Historically identified as a sensor of xenobiotics and mainly toxic substances, AhR has recently become an emerging pharmacological target in cancer, immunology, inflammatory conditions, and aging. Multiple AhR ligands are recognized, with plant occurring flavonoids being the largest group of natural ligands of AhR in the human diet. The biological implications of the modulatory effects of flavonoids on AhR could be highlighted from a toxicological and environmental concern and for the possible pharmacological applicability. Overall, the possible AhR-mediated harmful and/or beneficial effects of flavonoids need to be further investigated, since in many cases they are contradictory. Similar to other AhR modulators, flavonoids commonly exhibit tissue, organ, and species-specific activities on AhR. Such cellular-context dependency could be probably beneficial in their pharmacotherapeutic use. Flavones, flavonols, flavanones, and isoflavones are the main subclasses of flavonoids reported as AhR modulators. Some of the structural features of these groups of flavonoids that could be influencing their AhR effects are herein summarized. However, limited generalizations, as well as few outright structure-activity relationships can be suggested on the AhR agonism and/or antagonism caused by flavonoids.

## 1. Introduction

Flavonoids are the largest class of phenolic compounds found in nature, particularly important in the plant kingdom. In multiple plants, flavonoids are responsible for the color and flavor [1]. The aglycone and glycoside (i.e., glucosides, rhamnoglucosides, and rutinosides) forms of flavonoids are found in the human daily diet like fruits, vegetables, grains, seeds, bark, herbs, roots, flowers, stems, and spices. The natural distribution of these plant secondary metabolites, their inherent structural diversity, chemical properties, and ability to interact with biological systems modifying multiple target receptors in mammalians, have attracted attention during decades to their study in pharmacological and toxicological contexts [2,3]. 

Among the wide range of biological properties referable to flavonoids are included antioxidant, anti-inflammatory, antitumor, antiviral, hypolipidemic, antithrombotic, estrogenic, and antiallergic activities. Moreover, numerous pre-clinical and epidemiological studies reveal that flavonoids may exert protective effects against chronic conditions such as cancer, diabetes, and cardiovascular and neurodegenerative diseases, supporting the concept of a beneficial role for dietary flavonoids in human health [4,5,6,7]. 

The cellular detection of chemical signals from exogenous or endogenous sources can occur through chemoreceptors that commonly have cytoplasmic or transmembrane locations. The cytosolic chemosensor AhR is an evolutionarily conserved protein and a pleiotropic transcription factor with a key role in both health and diseases [8,9]. The signaling pathways of the formerly known as “dioxin receptor” have explained the mode of action of highly toxic chemicals, particularly 2,3,7,8-tetrachlorodibenzo-p-dioxin (TCDD) and derivatives [10]. Recently, there is increasing attention on AhR as an attractive target in cancer, immunology, inflammatory bowel diseases, and aging [11]. However, much about the molecular mechanisms and the activation/inactivation patterns of AhR upon ligand binding is pending closer analyses [12].

Multiple and structurally diverse chemicals including polyhydroxyalkanoates, polychlorinated biphenyls, bisphenols, polyphenols, benzothiazoles, and triarylmethanes have been evaluated as AhR ligands [13,14,15]. Plant flavonoids are considered the main group of natural dietary ligands of the AhR [16,17]. Hence, several well-known activators and inhibitors of AhR-mediated effects are flavonoids, with the flavonol quercetin being a prototypical representative. Depending on the cell context, quercetin has shown agonist and/or antagonist activities on AhR transcriptional activation [18]. This could suggest selective AhR modulation in which agonism or antagonism must be confirmed, as it is not clearly predictable [19].

This literature review provides a summary and update of the most recent state of the art on AhR and its natural flavonoid modulators. First, a detailed introduction is given on AhR’s molecular characteristics and signaling pathways, including the main physiological effects associated with its transcriptional activity. Next, plant occurring flavonoids that have been evaluated as AhR modulators, and their AhR agonism and/or antagonism, are presented. Finally, some chemical-structural features and substitution patterns of flavonoids that could be contributing to their AhR activity, are suggested. 

## 2. Aryl Hydrocarbon Receptor

### 2.1. Functional Domains

**Figure 1:**
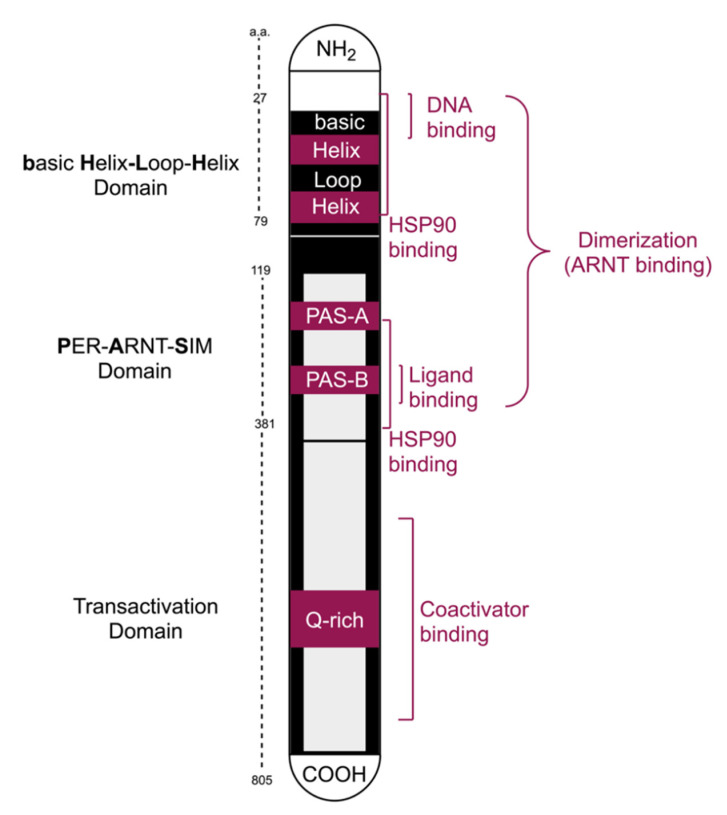
Schematic domain organization of the aryl hydrocarbon receptor (AhR) and its binding sites.

The transcription factor AhR was originally discovered in hepatocytes in the 1970s. Half a century later, multiple studies have confirmed that AhR signaling affects virtually every animal cell-type and organ in vertebrates and in many invertebrates [8]. The highest AhR expression has been reported in the liver, kidney, thymus, lung, spleen, and placenta [20]. Moreover, AhR is constitutively allocated in the nucleus of aggressive malignancies, where its target genes are widely expressed [21]. Figure 1 is a schematic representation of the AhR functional domains and binding sites.

The AhR and its nuclear translocator (ARNT) belong to the bHLH-PAS superfamily of transcription factors, the acronym for basic Helix-Loop-Helix and Periodic (PER)-ARNT-Single minded (**S**IM) [22]. Protein subfamilies may be stratified into two classes, with Class I including bHLH-PAS proteins such as the AhR, hypoxia-inducible factors, clock circadian regulators, and neuronal PAS protein, while Class II includes the ARNT subfamily of proteins located inside the nucleus and can heterodimerize with the cytosolic Class I members such as AhR. Both AhR and ARNT include an (N-) terminal bHLH domain, two PAS domains named A and B, and a transactivation domain carboxy (C-) terminal [23,24]. The PAS A domain controls dimerization through its connector helix and strengthens the binding to a distinct DNA motif in which bHLH plays the main role. With a three-loop insertion difference from PAS A, the PAS B is the AhR ligand-binding domain (LBD) [25]. In the transactivation domain, the Q-rich (Glutamine-rich) subdomain is the key region for transcriptional activation of xenobiotic response elements (XRE) in the DNA [26]. The binding to coactivators during the transcriptional process regulates the wide diversity and tissue specific AhR effects [27].

### 2.2. Cytoplasmic Complex and Signaling Pathways

**Figure 2:**
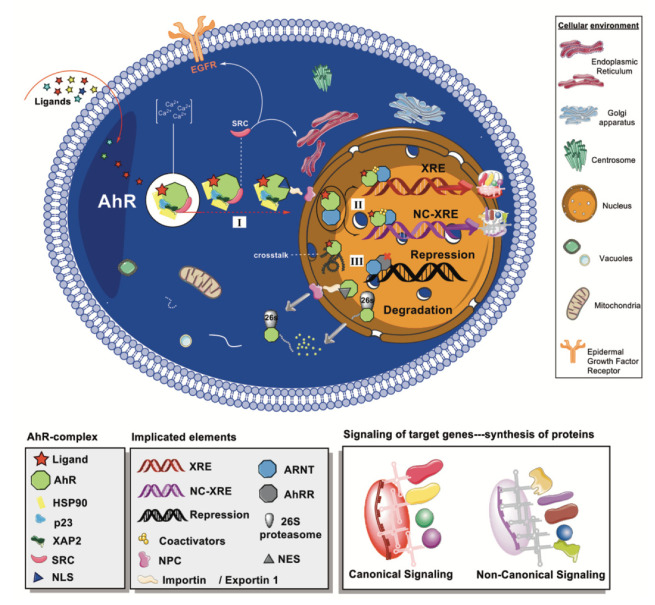
Schematic representation of AhR-mediated pathways. Ligands bind to the cytoplasmic AhR, where it forms a complex with HSP90, p23, XAP2, and SRC. (I) Agonist ligands promote conformational changes and the exposure of NLS, allowing the nuclear translocation mediated by importin β through the NPC. (II) Once in the nucleus, the heterodimer AhR-ARNT can bind to different sequences in the DNA and trigger the synthesis of proteins (e.g., CYP1A1, UGT1A6), which is additionally regulated by coactivators. (III) Crosstalk interactions with other signaling pathways are acknowledged (e.g., ESRs, AR). Moreover, the Ah receptor can be repressed by AhRR, and its degradation is mainly mediated by 26S proteasome.

The cytoplasmic inactive form of AhR (monomer) is found to be associated with two molecules of the 90-kDa heat shock protein (HSP90) chaperone, a co-chaperone p23, a termed X-associated protein 2 (XAP2), and a protein kinase SRC. While one of the HSP90 interacts with both the bHLH and PAS regions, the other unit is only bound to the PAS domain [28]. The HSP90 is a protein folding tool found also in steroid receptors such as estrogen, progesterone, and glucocorticoid receptors. The resistance to salt treatment and the absence of molybdate led to suggestions that HSP90/AhR binding may be the most stable binding known for that chaperone. However, inter-species differences for HSP90/AhR binding have been identified [29,30]. The co-chaperone p23 is a commonly found protein in HSP90 complexes, where it contributes to the ligand binding and to the release from HSP90 chaperone machinery [31,32]. More recently, a protecting role of p23 against AhR’s degradation independent of HSP90′s functions has been acknowledged [33]. Meanwhile, XAP2 presents a discrete domain organization with a tetratricopeptide repeat motif whose carboxyl-terminal domain is responsible for the interaction with AhR protein. In the AhR core complex, XAP2 positively or negatively modulates the receptor’s sensitivity to ligands and the transcriptional responses [34,35,36]. The c-SRC tyrosine kinase contributes to ligand recognition and indirectly ensures the transcriptional process [37]. Figure 2 represents some of the molecular consequences upon ligand binding to the AhR complex.

Genomic and non-genomic pathways are suggested for AhR signaling, as represented in Figure 2. The genomic induction of target genes is the fundamental and best-known mechanism of AhR-mediated signaling. The genomic pathway (I) starts once AhR agonists bind to the LBD, causing conformational changes in the PAS A that trigger the exposure of nuclear localization sequences (NLS) [38,39]. The SRC is then released from the AhR-chaperone complex to the cytosol, triggering phosphorylation processes [40]. The entrance to the nucleus is facilitated by importins that recognize the NLS. Specifically, importin β is the direct mediator that facilitates the passage to the cytoplasmic face of the nuclear pore complex (NPC) [41].

After the AhR-chaperone complex is translocated to the nucleus, the receptor heterodimerizes with ARNT, losing the chaperone proteins in the process and forming the AhR/ARNT complex (II in Figure 2) [42]. Depending on the target genes involved in the subsequent transcriptional activity triggered by the AhR/ARNT, the genomic signaling is classified as canonical when it is mediated by XRE or else as non-canonical or no consensus XRE (NC-XRE) responses (Figure 2). The canonical signaling starts when the AhR/ARNT complex binds to the XRE identified by the DNA consensus motif sequence 5′-TNGCGTG-3′ [43,44]. The NC-XREs, sometimes referred to as AhR Responsive Elements-II, are recognized by the consensus promoter region 5′-CATG{N6}C[T|A]TG-3′ [45]. A scaffold is formed upon binding to AhR core regions, inducing the recruitment of multiple coactivators that ultimately regulate the transcriptional process [27]. In addition, similar to other PAS proteins involved in critical signaling functions, AhR expression is controlled by a transcriptional repressor (AhRR) with a similar N-terminal region sequence [46]. Thus, the AhRR is an AhR competitor in that it may form the heterodimeric AhRR/ARNT complex, which binds to the XRE, and consequently repress the transcription. The AhRR effects mediate key processes such as inflammation and tumor growth that have led to its consideration as a drug target [47,48]. The repression pathway is also controlled by the receptor itself and its transcriptional outcomes forming a regulatory circuit [49].

The prototypical canonical target gene identified for AhR determines the induction of cytochrome P450 from family 1, subfamily A, polypeptide 1 (CYP1A1) xenobiotic- metabolizing enzyme [50,51]. At the same time, AhR regulates the basal *CYP1A1* expression through elements such as the so-called special protein family, and particularly the specificity protein 1 [52]. Moreover, AhR signaling through XRE has been associated with other family members such as CYP1A2 and CYP1B1 as well as phase II enzymes such as the UGT1A6 [53,54]. The understanding of the AhR complexity has been further expanded with the discovery of multiple nonclassical target genes in the past decades [55]. Some of the most important NC-XRE pathways are related to the tumor suppressor Kruppel-like factor 6 and the recruitment of flanking target genes such as the plasminogen activator inhibitor 1 and the p21^cip1^ protein [56]. 

Among the non-genomic mechanisms that AhR activation could trigger is the above- commented phosphorylation induced by the c-SRC of multiple key protein substrates such as the epidermal growth factor receptor (EGFR) [57,58]. Furthermore, c-SRC, the mitogen-activated protein kinases, reactive oxygen species (ROS), and AhR-dependent paths have been recently associated with the apoptosis of neuronal cells through the stress of the endoplasmic reticulum [59]. The proteasome-mediated degradation of ubiquitinated proteins is another important non-genomic pathway of AhR activation, which has been linked to the inflammatory process [60,61]. Thus, AhR can act as a ligand-dependent ubiquitin protein ligase (E3), promoting the proteolysis of other transcription factors [62,63]. Furthermore, AhR controls the levels of intracellular calcium and the induction of enzymes such as the cytosolic phospholipase A2 and cyclooxygenase 2 [64,65].

On the other hand, AhR modulation intervenes in the signaling networks of many other crucial cellular entities [66]. The crossroad with estrogen receptors (ESRα and ESRβ) is among the best-studied and it has been shown to lead to transcriptional activation of estrogen-responsive gene promoters and the consequent estrogenic effects [67]. Besides, AhR has proven to interact with the canonical Wnt/β-catenin pathway possibly mediated by some other entities such as R-spondin 1 [68], which is an important element in cellular proliferation as well as in sexual organ’s development and differentiation [69,70]. Such an interaction associates the receptor with the regulation of organogenesis and other key biological processes such as cell polarity and migration attributed to the glycoproteins Wnts [71]. 

AhR crosstalk interactions include effects on the transforming growth factor-β/bone morphogenetic protein (TGFβ/BMP) pathway. TGFβ/BMP is a target of drugs currently in phase I or phase II clinical trials for the treatment of ovarian cancer, hepatocellular carcinoma, glioblastoma, and melanoma [72]. In addition, the subunit RelB of NF-ĸB transcription factor family has shown to be functionally associated with AhR-mediated transcription. The resulting RelB/AhR complexes play an important role as inducers of pro-inflammatory chemokines such as IL-8, and this crosstalk mechanism is critically involved in immunity, cell proliferation, and apoptosis [73,74].

Lastly, the downregulation of AhR is described as a rapid crucial attenuation of AhR effects on gene transcription. The proteolytic degradation is a terminal step mainly ubiquitin-mediated through the 26S proteasome. It could be triggered if the receptor complex has a misfolded conformation prior to entry to the nucleus. Otherwise, after the signaling processes, AhR is ubiquitinated and degraded inside the nucleus or transferred to the cytoplasm via the Exportin 1 (or CRM-1) that recognizes the nuclear export signals (NES). In the cytosol, AhR is targeted and degraded by 26S proteasome [75,76]. Recently, lysosomal degradation of AhR via chaperone-mediated autophagy has been identified in triple-negative breast cancer cells [77].

### 2.3. AhR Effects and Modulators

The ligand, cell, tissue-specific, and pleiotropic effects of AhR-mediated activity are still puzzling and, in many cases, poorly understood [78]. Human diet and gut microbiome are tremendous sources of AhR modulators that trigger complex and fundamental processes in health and diseases [79,80]. It is believed that at least the downstream effects of dietary AhR inducers fit the renowned quote of the father of toxicology Paracelsus that *“Sola dosis facit venenum”* (the dose makes the poison) [12]. However, the receptor’s occupancy and the persistence of modulators are suggested to be, in many cases, far more relevant than the dosage. Low dosage effects still are underestimated and not fully understood by current science. Overall, AhR pathways intricacies have led some to consider that many of its adverse health consequences could be an exacerbated cellular response to its endogenous roles [66,81]. The truth is that these contradictory statements remain to be clarified on this fascinating transcription factor [10].

Toxicologists have been for decades interested in AhR-dependent signaling as a key intermediate step in the toxic responses to dioxin-like compounds [10]. Immunological, hepatic, and endocrine disruptions, as well as carcinogenic effects, are attributed to AhR-mediated activity [55]. However, the proven AhR physiological functions, particularly important in barrier organs as the gut or the skin, are nowadays driving a new line of research for the formerly known as “dioxin receptor” [9]. 

AhR has been associated with key processes such as development, cell cycle regulation, immunity, resilience against stress, homeostasis, and metabolism [82]. Potential therapeutic uses of AhR modulation include maintenance of lung health [83], control of the inflammatory process in vascular tissues [84], treatment of liver and cystic fibrosis [85,86], control of the antioxidant response [87], and regulation of neural functions in both vertebrates and invertebrates [88]. 

**Figure 3:**
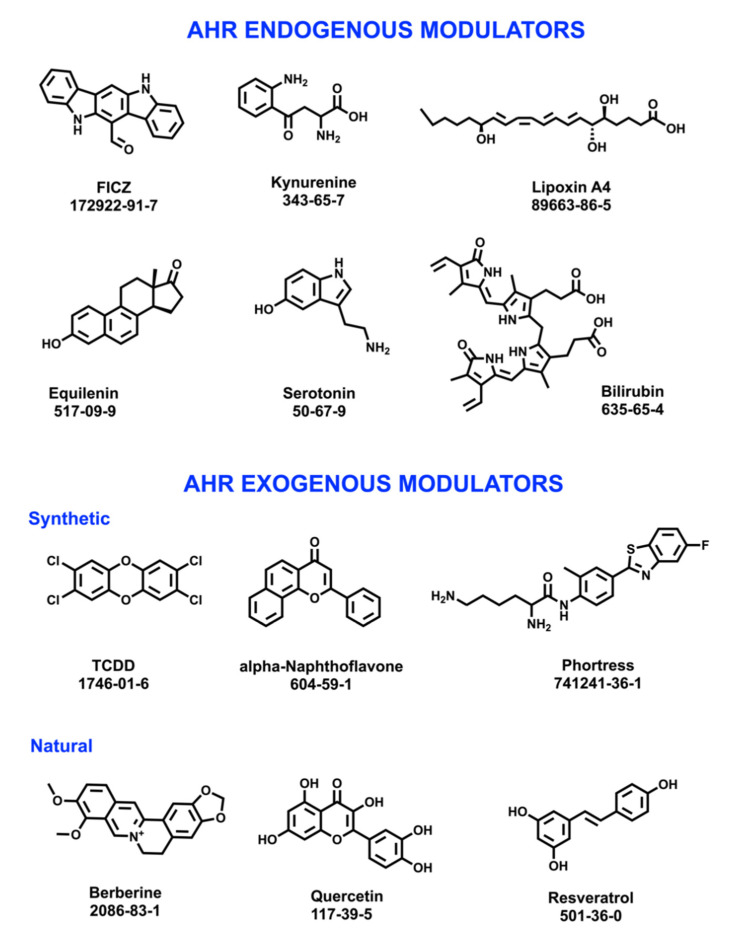
Examples of AhR endogenous and exogenous modulators (structures, common names, and CAS numbers are shown).

Numerous and structurally diverse chemical entities have been identified as AhR agonist and/or antagonist modulators (Figure 3). Hence, the receptor has been considered a “promiscuous” sensor of chemical entities [89]. Endogenous entities such as the tryptophan derivative 6-formylindolo [3, 2-b]carbazole (FICZ) [90] and the neurotransmitter serotonin [91], are AhR modulators. Exogenous compounds exhibiting AhR modulatory effects include the toxicant TCDD, the synthetic alpha-Naphthoflavone and the drug Phorthress, as well as naturally occurring compounds such as berberine, quercetin, and resveratrol.

Discovering chemical entities acting as AhR agonists and antagonists contribute to a better understanding of the molecular interactions as well as the toxicological and therapeutic roles of the receptor [92]. In toxicology, this has helped to identify potential high concern substances and environmental contaminants, particularly persistent organic pollutants whose exacerbated effects could trigger the adverse outcomes associated with AhR transcriptional activity [93].

Inflammatory and immunological diseases could be modulated through AhR expression and particularly those affecting gut and intestinal tissues [79,80,94]. Thus, promising drug candidates such as the designed AhR ligands NPD-0414-2 and NPD-0414-24, have been recently suggested in the therapy of colitis [95]. However, probably the most significant pharmacological applications of targeting AhR are in the treatment of malignancies. Hence, the chemotherapy of CYP1A1-positive tumors includes AhR agonists such as Phortress (NSC 710305) [96] and Aminoflavone (NSC 686288) [97], with demonstrated antiproliferative activity through AhR activation.

Some authors suggest the term of selective AhR modulators (SAhRMs) instead of common categorizations based on their binding affinity, structural similarities, origins, or toxicity. Such a consideration is based on the tissue, organ, and species-specific activities exhibited by most AhR ligands, but particularly by non-dioxin-like ligands such as flavonoids. Deeper investigations that focus on the selectivity patterns and the agonist and antagonist responses of AhR modulators could significantly contribute to their clinical applications and to the control of their toxicity [19].

## 3. Flavonoids: Generalities and Modulating Effects of AhR

Flavonoids are secondary metabolites widely distributed throughout the plant kingdom in both edible and non-edible species. Countless literature reports indicate a link between flavonoid’s consumption and health benefits (or risks), due to their antioxidant, antiproliferative, estrogenic, or anti-estrogenic properties [98,99]. As a large reservoir of chemical diversity, plant polyphenols are involved in a varied spectrum of gene and enzymatic regulation mechanisms [100,101,102].

**Figure 4:**
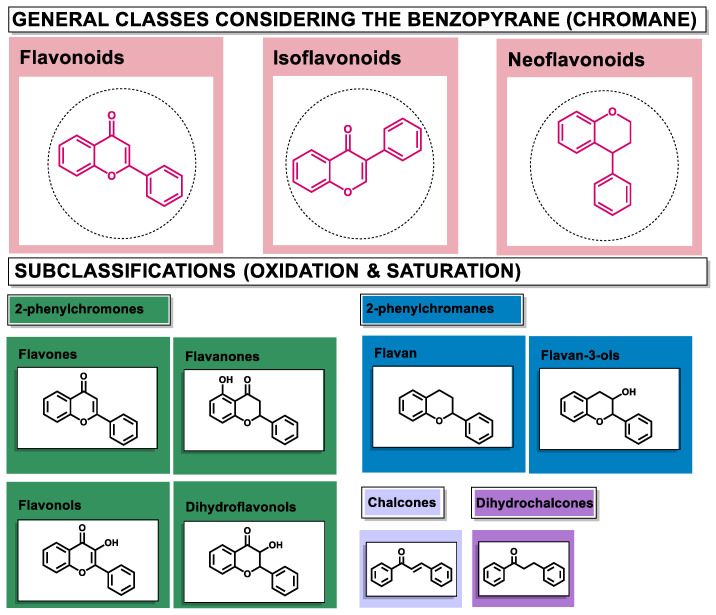
General classes of flavonoids and some relevant subclassifications.

Structurally, flavonoids are characterized by their basic skeleton arranged in the form C6-C3-C6, which corresponds to two benzene rings linked by a unit of three carbon atoms, which may or may not give rise to a third ring. Despite the various criteria regarding their classification, three general classes are recognized according to the linkage position of the aromatic ring with respect to the benzopyrane (chromane) moiety: flavonoids (2-phenyl-1,4-benzopyrones), isoflavonoids (3-phenyl-1,4-benzopyrones), and neoflavonoids (4-phenyl-1,2-benzopyrones) [101]. Moreover, they can be subclassified into subgroups according to the degree of oxidation of the pyranic ring, which can be opened and recycled in the furan cycle and linking chain unsaturation: 2-phenylchromones (flavones, flavonols, flavanones, and dihydroflavonols); 2-phenylchromanes (flavans, flavan-3-ols, flavan-3,4-diols), chalcones (represented in Figure 4), and some others such as anthocyanins (2-phenyl-benzopyrilium) and aurones [2,3,103,104].

Regarding their chemical reactivity, flavonoids occur as hydroxylated, methylated, and prenylated aglycones or glycosylated derivatives, either *O*-glycosides formed via linkage of the sugar unit to the hydroxyls, or C-glycosides directly to the carbon atom of the flavonoid skeleton [2,105]. In the cytosol (pH 7.4), most flavonoids form a mixture of phenolate anions and neutral phenols. Their proportion depends on the pK_a_ of each phenolic group in the structure. Since most flavonoids are weak hydrophobic acids, depending on their lipophilicity, they have the potential to cross cellular and mitochondrial membranes and act as protonophores. Sometimes even minimal structural modifications alter the solubility, reactivity, and stability of flavonoids that ultimately lead to their bioavailability differences and the diversity of biological effects across the groups [106,107].

The current uses of plant flavonoids cover cosmetic, nutraceutical, and pharmaceutical industries [3,108,109]. Their prophylactic efficacy against neoplastic [110,111,112], cardiovascular [113,114,115], bone [116], and neurological disorders [117] are among the most widespread. Pharmacological studies have revealed also anti-inflammatory [118,119] and antibacterial potential [102] of flavonoids, as well as their applications in the prevention and treatment of obesity [120] and diabetes [121,122]. Besides, antioxidant effects have been attributed to all flavonoid subclasses [7]. Thus, most of the anticancer properties ascribed to plants and natural extracts from traditional medicine are explained through flavonoids’ ability to scavenge free radicals and other ROS, modulate ROS-scavenging enzymes, chelate metal ions, and prevent oxidative stress-related conditions [104,123]. However, the mechanisms mediating the diverse bioactivity profiles of flavonoids, which are determined by minimal structural differences, still are pending further investigations [2,6].

A wide variety of flavonoids are suggested to be modulators of AhR function. The complexity of the role of AhR in physiology and in pathological conditions, lead to discrepancies in the positive or negative molecular consequences of the AhR transcriptional modulation caused by flavonoids [11,16,124]. Furthermore, it is recognized that beyond a dose dependence of food flavonoids when acting on AhR, these ligands could display different activities depending on the exposure time, and their effects are variable among species and tissues [125,126,127]. Table 1 shows the list of flavonoids studied as AhR modulators, the main bioassays used, and the common range of concentrations tested.

As presented in Table 1, in recent years, accurate and informative techniques such as RT-qPCR, LucRGA, ChIP, Co-IP, EROD, IF, IHC, and WB have been used to assess the modulatory capacity of flavonoids on AhR. Meanwhile, the tested concentrations are commonly between 10 μM and 100 μM. The main subclasses of flavonoids studied as AhR modulators are flavones, flavonols, flavanones, and isoflavones. In these four classes, agonist and/or antagonist activities, as well as inactivity on AhR-mediated effects are reported, as summarized in Table 2 (Flavones), Table 3 (Flavonols), Table 4 (Flavanones), and Table 5 (Isoflavones).

As shown in Table 2, Table 3, Table 4 and Table 5, generalizations of structure-activity relationships are not possible when studying flavonoids as AhR modulators. AhR agonism and antagonism and, in many cases, both effects have been reported for flavones, flavonols, flavanones, and isoflavones. Nevertheless, the following patterns emerge.

1. The modulation of AhR by aglycones and glycosides analogous does not seem to differ significantly, noticeable when compare flavone luteolin vs. luteolin 7,3′-diglucoside (Table 2), that both display blockage effect on AhR transcriptional activation, although luteolin also exhibited agonist effects. In flavonols, the prototypical AhR modulator quercetin is reported as an agonist and as an antagonist of the receptor (Table 3), while its glycoside analogous guaijaverin is described as agonist, quercitrin as antagonist, and rutin has shown both agonist and antagonist effects. The flavanone aglycone hesperetin is reported as AhR antagonist, while exhibited also AhR agonist effects as the glycoside hesperidin. Naringenin differs from naringin in the agonist capacity, reported only for the glycoside derivative (Table 4).
2. Flavone and flavonol analogues (Table 2 and Table 3) do not display substantial differences in their effects deduced when compare flavone vs. flavonol, apigenin vs. kaempferol, and luteolin vs. quercetin. Most of them have shown AhR agonism and AhR antagonism.
3. Comparing the flavone apigenin vs. the isoflavone analogous genistein, the modification from position 2 to position 3 appears to lead to the loss of agonist effects (Table 2 and Table 5). Similarly, a loss of agonism is observed when compared to unsaturated analogous (apigenin and kaempferol) vs. the saturated flavanone naringenin (Table 2, Table 3 and Table 4).
4. The hydroxy-analogous derivatives representing the four classes of flavonoids: apigenin kaempferol, naringenin, and genistein have displayed AhR antagonist activity (Table 2, Table 3, Table 4 and Table 5).
5. In flavone derivatives, the 7-methoxy substitution causes a loss of agonist capacity noticeable when compared genkwanin vs. the tri-hydroxy substituted apigenin (Table 2). In flavanones (naringenin vs. sakuranetin) (Table 4) and isoflavones (genistein vs. prunetin) (Table 5), 7-methoxy derivatives and 7-hydroxy derivatives are all reported as non-agonist of AhR. The 7-hydroxy flavanone naringenin and the 7-hydroxy isoflavone genistein have shown AhR antagonism.
6. The 4-methoxy substitution does not seem to affect the AhR agonist potential in flavones (acacetin vs. apigenin) (Table 2).
7. In isoflavones, 4′-methoxy substitution confers AhR agonist activity (daidzein vs. formononetin and biochanin A vs. genistein) (Table 5).
8. Compounds 4′,5,7-trimethoxy flavone (Table 2) and 4′,5,7-trimethoxy flavanone (Table 4) both lack AhR agonist effects. However, the analogous 4′,5,7-trimethoxy isoflavone (Table 5) is an AhR agonist.
9. The 4′,7-dimethoxy substitution is positive for AhR agonism in flavones compared to 4′,5,7-trimethoxy substitution (Table 2). The opposite occurs in isoflavones (Table 5).
10. The 3′,4′,5,7-tetramethoxyflavone is AhR agonist (Table 2), but the analogous 3′,4′,5,7-tetramethoxyisoflavone does not activate the receptor (Table 5).
11. Most of the prenylated flavonoids are described as AhR agonists, such as the flavonol icaritin (Table 3) and the flavanones 6-prenylnaringenin and 8-prenylnaringenin (Table 4). However, this is not the case of the flavanone isoxanthohumol (Table 4).

Historically, the effect of flavonoids on AhR was associated with their capacity to suppress the receptor transcriptional activity [150]. However, as demonstrated in this review, the range of AhR agonist and antagonist effects of flavonoids have been shown to be much more complex and diverse than previously thought. The analysis of the structural substitution patterns of flavonoids does not appear to lead to general predictions of their potential AhR modulation. Differences in bioassays and experimental conditions and laboratories could be playing an important role in the AhR activity profile reported in the literature for flavonoids. Furthermore, as previously mentioned, several AhR, including some flavonoids, are considered to be SAhRMs. That is, the type of tissue and organ in which the AhR effects caused by these ligands are evaluated largely determines their behavior on the receptor [19,151]. Such selectivity on AhR could probably contribute to the therapeutical applicability of flavonoids as AhR mediators, as it has been suggested for the isoflavones biochanin A and formononetin used to ameliorate menopausal complaints [151]. However, the complexity and broad-based evidence required to ensure optimal ligand effects, in the absence of readily predictive AhR agonism and/or antagonism, is a matter that should be extensively studied. Therefore, further investigations on AhR transcriptional modulation caused by flavonoids need to be conducted in order to regulate their harmful consequences and get the most of their therapeutical potential through this pathway.

## 4. Conclusions

Despite several decades of research on flavonoid derivatives, these ubiquitous constituents of food remain surprising for their bioactivity profiles. AhR is a pleiotropic chemosensor with toxicological and pharmacological implications. Therefore, AhR modulation caused by flavonoids could impact several fields including food science, nutrition, drug discovery, agronomy, and environmental research. Basic research on AhR and its multiple modulators, including the diet-incorporated flavonoids, could provide useful premises to health care, adequate nutrition, and risk assessment in the translational science paradigm.

## 5. Search Methodology

The data collected in this review article aimed to concisely summarize the structural characteristics and functions of the transcription factor AhR (from 1990–2020), as well as to identify all plant flavonoids that have been evaluated as AhR modulators (from 2000–2020). The most common and accurate sources were used to run the search including the scientific databases PubMed, Scopus, Web of Science, Science Direct, and Google Scholar. Keywords used to carry out the search included “dioxin receptor”, “ah receptor”, “aryl hydrocarbon receptor”, and “flavonoid”. The search syntaxes for the different databases were considered. Results of the search strategies implemented independently by two researchers were contrasted.

## Figures and Tables

**Table 1:** Plant occurring flavonoids studied as aryl hydrocarbon receptor (AhR) modulators through different bioassays.

Flavonoids	Bioassays *^a^*	Cell lines & Tissues *^b^*	[μM]	Reference
**Flavones** Acacetin, Apigenin, Baicalin, Chrysin, Flavone, Genkwanin, Luteolin, Luteolin 7,3′-diglucoside, Scutellarein, Tangeretin, Tricetin, 4′,7-dimethoxy-5-hydroxyflavone, 4′,5,7-trimethoxyflavone, 3′,4′,5,7-tetramethoxyflavone.	RT-qPCR, PCR, LucRGA, ChIP, WB, IF, IHC	Caco2, YAMC, DLN, HepG2 AhR-Lucia, H1L6.1c2, 3T3-L1 (AhR) HCT116, MDA-MB-231, Hepatic tissue (rats), Myocardial tissue (mice)	10–100	[128,129,130,131,132,133,134,135,136,137]
**Flavonols** Fisetin, Flavonol, Galangin, Gossypetin, Guaijaverin, Icaritin, Isorhamnetin, Kaempferol, Morin, Myricetin, Quercetin, Quercitrin, Robinetin, Rutin. Tamarixetin, 3,6,2′,3′-Tetrahydroxyflavone, 3,6,2′,4′-Tetrahydroxyflavone.	RT-qPCR, ChIP, WB, LucRGA	LNCaP, CWR22Rv1, Caco2, H1L6.1c2, 3T3-L1 (AhR), PBMEC/C1-2, HepG2 AhR-Lucia	10–100	[128,129,135,136,138,139]
**Dihydroflavonols** Taxifolin, Dihydromyricetin	RT-qPCR, ChIP, WB, LucRGA	Caco2, HepG2	0.1–100	[135,140]
**Flavolignane** Silymarin	LucRGA	H1L6.1c2	25	[139]
**Flavanones** Alpinetin, Eriodictyol, Flavanone, Hesperetin, Hesperidin, Isoxanthohumol, Naringenin, Naringin, Naringenin Trimethyl Ether, Sakuranetin, 6-Prenylnaringenin, 8-Prenylnaringenin	LucRGA, RT-qPCR, WB, ChIP, Co-IP, EROD	HepG2, MCF-7, HepG2 AhR-Lucia, H1L6.1c2, Caco2, YAMC, PBMEC/C1-2, EL-4	0.1–100	[136,137,139,141,142]
**Flavan 3-ol** Epigallocatechin gallate	WB, LucRGA	3T3-L1 (AhR), HepG2 AhR-Lucia	30–100	[128,136]
**Chalcone** Cardamonin	qPCR, WB	THP-1	3.0–30	[143]
**Isoflavones** Biochanin A, Daidzein, Formononetin, Genistein, Puerarin, Prunetin, 4′,7-dimethoxy-5-hydroxyisoflavone, 4′,5,7-trimethoxyisoflavone, 3′,4′,5,7-tetramethoxyisoflavone	RT-qPCR, LucRGA, WB	Caco2, YAMC, H1L6.1c2, MCF-7, HC-04, HepG2 (AZ-AhR), Hepa-1c1c7, HepG2 AhR-Lucia	0.1–1000	[136,137,139,144,145,146]

***^a^*** Bioassays: Chromatin immunoprecipitation (ChIP), Co-immunoprecipitation (Co-IP), EROD, ethoxyresorufin-O-deethylase (EROD), Immunofluorescence (IF), Immunohistochemical (IHC), Luciferase reporter gene assay (LucRGA), reverse transcriptase-quantitative polymerase chain reaction (RT-qPCR), western blotting (WB). ***^b^*** Cell Lines: draining lymph node (DLN), human breast adenocarcinoma (MCF-7), human colon cancer (HCT116), human epithelial colorectal adenocarcinoma (Caco2), human hepatocyte (HC-04), human hepatoma (HepG2), human monocytic leukemia (THP-1), human prostate carcinoma (LNCaP, CWR22Rv1), mouse adipocytes (3T3-L1), mouse colonic epithelium (YAMC), mouse hepatoma (H1L6.1c2, Hepa-1c1c7), mouse lymphoblast (EL-4), porcine brain microvascular endothelium (PBMEC/C1-2).

**Table 2:** Structures and AhR activity reported for the flavone class of compounds.

Flavones			
Flavone 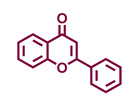	Chrysin 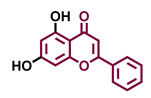	Apigenin 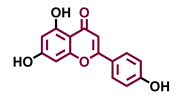	Luteolin 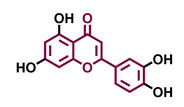
Agonist [139] Antagonist [129]	Agonist [133,139] Antagonist [129]	Agonist [135,136,139] Antagonist [129]	Agonist [135] Antagonist [147]
Tricetin 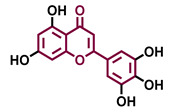	Scutellarein 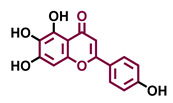	Acacetin 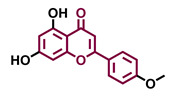	Genkwanin 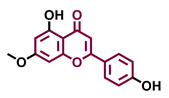
Agonist [135]	Agonist [136]	Agonist [137]	Non-agonist [137]
4′,7-Dimethoxy-5-hydroxyflavone 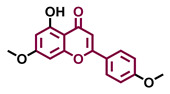	4′,5,7-Trimethoxyflavone 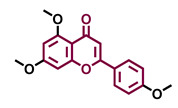	3′,4′,5,7-tetramethoxyflavone 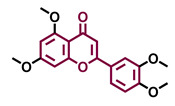	Tangeretin 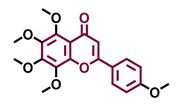
Agonist [137]	Non-agonist [137]	Agonist [137]	Antagonist [132]
Baicalin 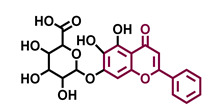		Luteolin 7,3′-diglucoside 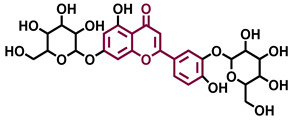	
Agonist [131,139] Antagonist [130]		Antagonist [128]	

**Table 3:** Structures and AhR activity reported for the flavonol class of compounds.

Flavonols			
Flavonol 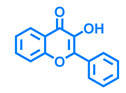	Galangin 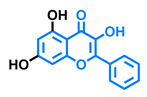	Kaempferol 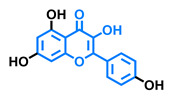	Fisetin 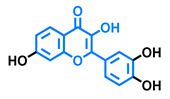
Agonist [128] Antagonist [128]	Antagonist [128]	Agonist [135] Antagonist [128,135]	Agonist [135,139] Antagonist [128,135]
3,6,2′,3′-Tetrahydroxyflavone 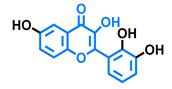	3,6,2′,4′-Tetrahydroxyflavone 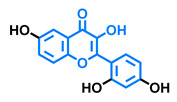	Morin 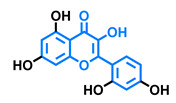	Quercetin 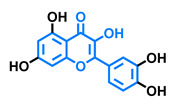
Agonist [135]	Agonist [135] Antagonist [135]	Agonist [135] Antagonist [129]	Agonist [135,139] Antagonist [128]
Robinetin 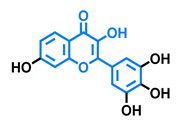	Gossypetin 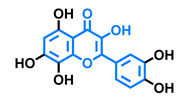	Myricetin 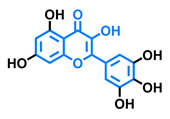	Isorhamnetin 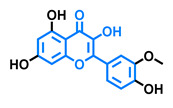
Agonist [135]	Agonist [135]	Agonist [135] Antagonist [129]	Antagonist [129]
Tamarixetin 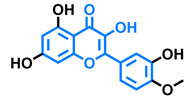	Icaritin 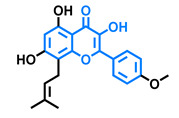	Guaijaverin 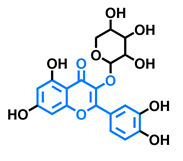	Quercitrin 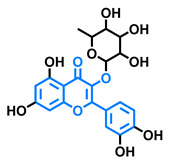
Agonist [128], Antagonist [129]	Agonist [138]	Agonist [136]	Antagonist [128]
	Rutin 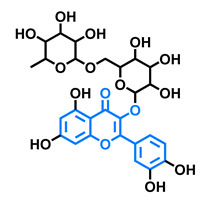 Agonist [139] Antagonist [128]		

**Table 4:** Structures and AhR activity reported for the flavanone class of compounds.

Flavanones		
Flavanone 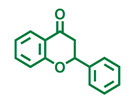	Naringenin 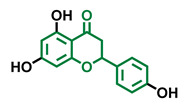	Eriodictyol 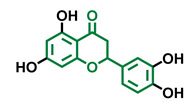
Antagonist [129] Agonist [128]	Antagonist [129] Non-agonist [137]	Antagonist [128]
Alpinetin 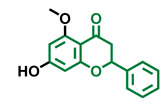	Sakuranetin 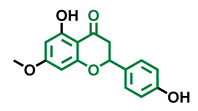	Hesperetin 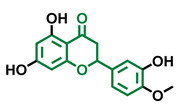
Agonist [148,149]	Non-agonist [136]	Agonist [139] Antagonist [142]
Naringenin Trimethyl Ether 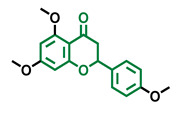	Hesperidin 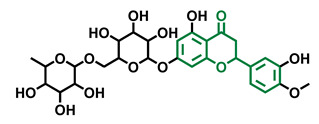	Naringin 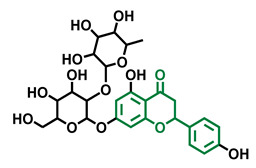
Non-agonist [137]	Agonist [136,139]	Agonist [139] Antagonist [128]
6-Prenylnaringenin 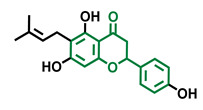	8-Prenylnaringenin 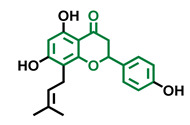	Isoxanthohumol 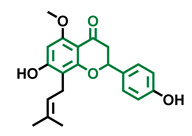
Agonist [141]	Agonist [141]	Non-agonist [141]

**Table 5:** Structures and AhR activity reported for the isoflavone class of compounds.

Isoflavones			
Daidzein 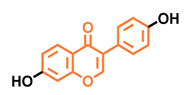	Genistein 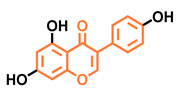	Formononetin 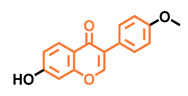	Biochanin A 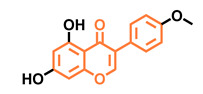
Antagonist [144] Non-agonist [145]	Antagonist [144,146] Non-agonist [136,137]	Agonist [144,145]	Agonist [139,144,145]
Prunetin 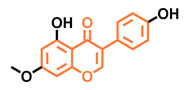	4′,7-dimethoxy-5-hydroxyisoflavone 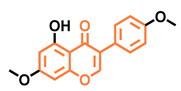	4′,5,7-trimethoxyisoflavone 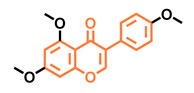	3′,4′,5,7-tetramethoxyisoflavone 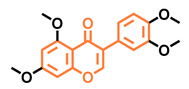
Non-agonist [137]	Non-agonist [137]	Agonist [137]	Non-agonist [137]
	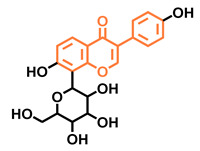		Puerarin
	Non-antagonist [128]		

## Data Availability

Not applicable.
